# Microfluidics as tool to prepare size-tunable PLGA nanoparticles with high curcumin encapsulation for efficient mucus penetration

**DOI:** 10.3762/bjnano.10.220

**Published:** 2019-11-19

**Authors:** Nashrawan Lababidi, Valentin Sigal, Aljoscha Koenneke, Konrad Schwarzkopf, Andreas Manz, Marc Schneider

**Affiliations:** 1 Department of Pharmacy, Biopharmaceutics and Pharmaceutical Technology, Saarland University, 66123 Saarbrücken, Germany; 2 Department of Anaesthesia and Intensive Care, Klinikum Saarbrücken, Winterberg, 66119 Saarbrücken, Germany; 3 KIST Europe, 66123 Saarbrücken, Germany

**Keywords:** curcumin, human pulmonary mucus, microfluidics, muco-penetrating nanoparticles, nanomedicine, permeability, PLGA nanoparticles

## Abstract

Great challenges still remain to develop drug carriers able to penetrate biological barriers (such as the dense mucus in cystic fibrosis) and for the treatment of bacteria residing in biofilms, embedded in mucus. Drug carrier systems such as nanoparticles (NPs) require proper surface chemistry and small size to ensure their permeability through the hydrogel-like systems. We have employed a microfluidic system to fabricate poly(lactic-*co*-glycolic acid) (PLGA) nanoparticles coated with a muco-penetrating stabilizer (Pluronic), with a tunable hydrodynamic diameter ranging from 40 nm to 160 nm. The size dependence was evaluated by varying different parameters during preparation, namely polymer concentration, stabilizer concentration, solvent nature, the width of the focus mixing channel, flow rate ratio and total flow rate. Furthermore, the influence of the length of the focus mixing channel on the size was evaluated in order to better understand the nucleation–growth mechanism. Surprisingly, the channel length was revealed to have no effect on particle size for the chosen settings. In addition, curcumin was loaded (EE% of ≈68%) very efficiently into the nanoparticles. Finally, the permeability of muco-penetrating PLGA NPs through pulmonary human mucus was assessed; small NPs with a diameter of less than 100 nm showed fast permeation, underlining the potential of microfluidics for such pharmaceutical applications.

## Introduction

In the last decades, the application of nanotechnology in medicine has gained significant attention, especially in the biomedical field for vaccine delivery [1–2], in anticancer therapies [3–4], as well as for gene delivery [5–6]. Owing to the unique physicochemical properties of nanoparticles, the nanoparticle surface can be specifically modified to meet the needs of the desired application [7–8]. Such surface modifications can also be applied to protect drug carriers from being inactivated by avoiding interaction with mucus [9–11]. Nanoparticles (NPs) have shown a tremendous effect in terms of facilitating the diffusion of drugs through biological barriers, for example, through thick mucus in cystic fibrosis [10,12–16]. Notably, only NPs with size less than 200 nm have the ability to permeate easily through mucus without being immobilized by the natural size-filtering mechanism [10,17–19]. Furthermore, modifying the surface chemistry of NPs is beneficial for avoiding the interaction/filtering mechanisms such as H-bond interaction and electrostatic interactions [15,17,20–24]. Moreover, NPs as a carrier system have shown the ability to protect the loaded drug from inactivation, reduce unwanted side effects and enhance the efficacy of the active pharmaceutical ingredient (API) due to improved solubility and bioavailability [25]. As penetrating particulate systems also raise the question of toxicity, biocompatible systems such as poly(lactide-*co*-glycolide) (PLGA) (a very benign material) are considered to be well-suited. PLGA NPs have been extensively studied in the pharmaceutical field, relying on PLGA's biodegradability and the fact that it is FDA approved for some products [26–27]. Many different methods have been established to prepare PLGA NPs, such as double emulsion and nanoprecipitation [28–29]. Among many other techniques, nanoprecipitation was adopted very quickly to prepare sub-micrometer particles, because it is a simple and straightforward technique, without the involvement of any chemical additives, and also does not require harsh formulation parameters, such as high energy input or mechanical shear stress (e.g., by sonification) [30–31]. Nonetheless, the preparation of sub-micrometer NPs in a conventional “bench-top” nanoprecipitation method still faces several critical challenges, such as the lack of reproducibility, which restricts it from being widely adopted in the pharmaceutical industry [32–33]. This issue is mainly attributed to the poor control of the mixing time in many approaches. This problem holds true especially for the preparation of NPs of size less than 200 nm, which are preferred with respect to their biological penetration potential. Improved control with respect to the mixing time can be achieved by utilizing impinging jets or microfluidic systems, as they allow to control the mixing time on the order of milliseconds instead of minutes [34–35]. The mixing time was proven to be the major factor that influences the size and monodispersity of colloidal particles [36]. The mixing time can be tuned by adjusting the flow rate of the solvents or channel geometry. Additionally, fast mixing (as in microfluidics) has shown a variety of advantages over conventional methods (bench-top) regarding the physicochemical and encapsulation properties of the nanoparticles [36]. A LabSmith system (LabSmith, Inc., Livermore, USA) was used for the manufacture of PLGA NPs using the nanoprecipitation method. This system offers stable conditions to produce monodisperse particles of small size. At the same time, it offers the possibility to vary some additional factors during preparation, such as channel diameter and channel length [37]. For the successful permeation of the particles through mucus, small particles are required. To ensure proper treatment, it is necessary to reach the target dose; therefore, a high drug loading into the NP carrier is necessary to compensate for their small size in order to reach the target dose. In this study, the encapsulation of a lipophilic model drug (curcumin, a nonsteroidal naturally anti-inflammatory drug) was assessed by comparing different preparation approaches, such as bench-top preparations with different injection procedures. The choice of an anti-inflammatory drug for potential loading into PLGA NPs was made to address strong and continuous inflammatory responses which could have an impact in, for example, cystic fibrosis treatment [38]. The potential for application in cystic fibrosis treatment was highlighted monitoring the penetration of the particles through human pulmonary mucus.

## Results and Discussion

### Influence of different parameters on the NP size

Smaller nanoparticles (NPs) are known to have a better diffusion through mucus, whereby they can evade the natural size-filtration mechanism [10,14,39]. Furthermore, the surface chemistry of NPs plays a crucial role in facilitating their penetration through mucus [9]. In this context, we have used microfluidics to produce size-tunable PLGA NPs coated with a muco-penetrating stabilizer (Pluronic F68). Relevant factors influencing the NP size were examined, such as flow rate ratio, PLGA concentration, solvent type, diameter of the mixing channel, and stabilizer concentration. Furthermore, as our system allows us to cut different lengths of mixing channels, the impact of the length of the mixing channel on the nucleation–growth mechanism of the nanoprecipitation process was evaluated, which is an underestimated aspect of current microfluidic research.

### Effect of flow rate ratio and total flow speed

The flow rate ratio was calculated as

[1] $$\text{Flow rate ratio}=\frac{\text{flow rate of organic phase}}{\text{flow rate of aqueous phase}}$$

**Figure 1:**
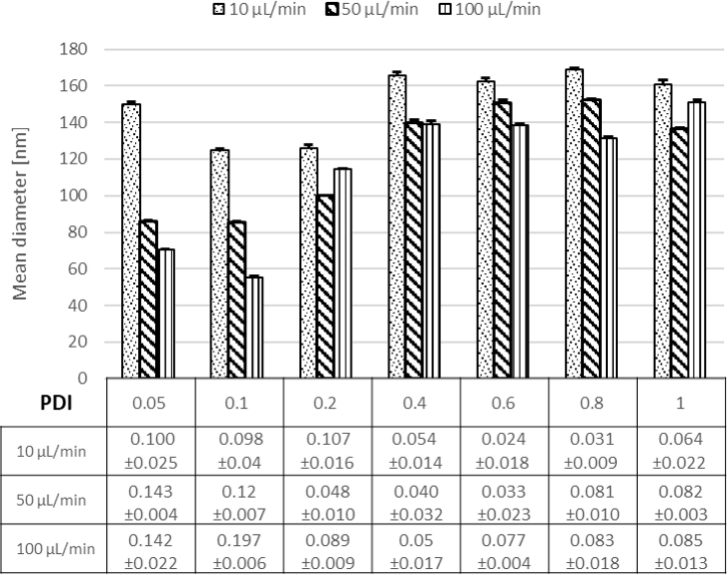
The effect of the flow rate ratio (0.05–1.0) and flow speed of the aqueous phase (10–100 µL/min) on NP size and size distribution.

We varied the flow rate ratio (FRR) of the organic phase to the aqueous phase from 0.05–1 and the flow speed of the aqueous phase was set to a fixed value ranging from 10 to 100 µL/min while adapting the organic phase volume accordingly. At a flow rate ratio of 0.05, a substantial reduction of the NP size from ≈150 to 70 nm was obtained (Figure 1).

**Figure 2:**
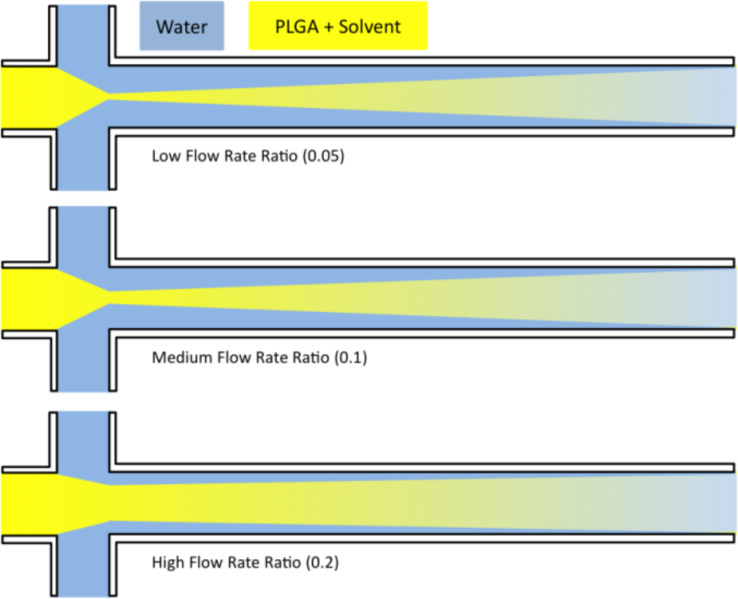
Illustration the impact of increasing the flow rate ratio on the mixing pattern and the geometry of the flowing solvents. The increasing mixing time at higher ratios could lead to larger particle sizes.

The reduction in the NP size is attributed to the rapid and efficient mixing process as described in literature [36]. Further, the Ostwald ripening phenomena could be avoided at short mixing times [40]. Additionally, for FRRs above 0.2 (by adjusting only the flow rate of the organic phase) larger NPs were obtained. This implies that an increase in the width of the focus point of the organic phase occurred as a result of the higher FRR as illustrated in Figure 2 [41]. For this reason, a longer time was required for the diffusive material to be mixed. Another scenario was proposed by Wang et al. [42], who suggested that the increase in NP size at higher FRR is related to the use of larger amounts of solvent causing swelling of the NPs. Afterwards, the influence of increasing the flow speed was studied when using higher flow speeds of the aqueous phase (but adjusting the same FRR) on the size of the NPs. The aqueous phase flow was set to 10, 50 or 100 µL/min while keeping the flow of the organic phase constant at 10 µL/min. It was observed that an increase in the flow speed of the aqueous phase from 10–100 µL/min led to a reduction in the mean diameter of the NPs from 150 to 70 nm at a flow ratio of 0.05 (Figure 1). This evidence points to the fact that most likely the polymer concentration decreases by increasing the aqueous phase flow rate, thus small NPs were obtained. Another reason could be that the rate of the NP growth decreased as well [43–44]. Markedly, the tendency of NP aggregation decreased at a higher flow of the aqueous phase due to the large volume of the aqueous phase, which prevents particle interaction (this was indicated by PDI (polydispersity index, respreswenting the size distribution) values always being <0.1 for all particles prepared by microfluidics). Moreover, the particles are easier to redisperse and are more stable within the aqueous suspension.

### Effect of polymer concentration

**Figure 3:**
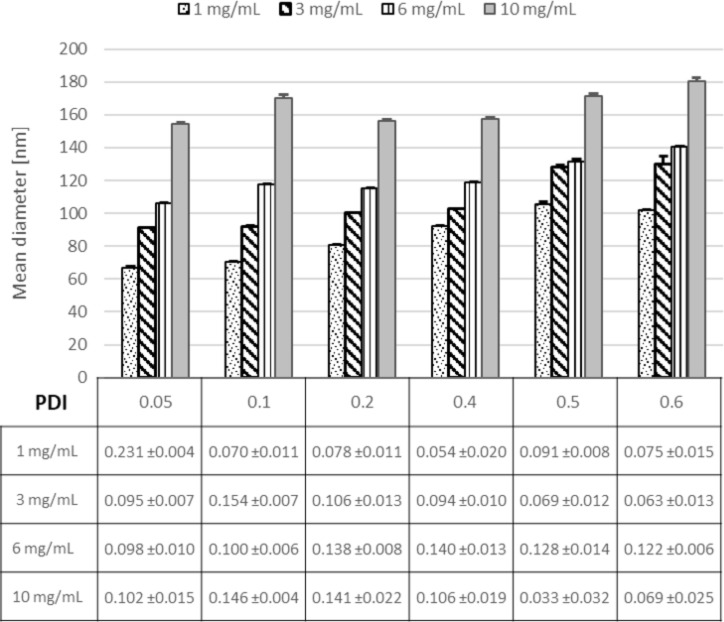
Effect of different PLGA concentrations (1–10 mg/mL) on NP size and PDI at different flow rate ratios.

The nanoprecipitation mechanism is predicted to be primarily ruled by the Marangoni effect where the concentration gradient and the concentration of the polymer play a role in influencing the colloidal properties [45]. For this purpose, the influence of the polymer concentration (PLGA) on the NP mean diameter was tested. It can be observed that by varying the polymer concentration (PLGA) from 1 mg/mL up to 10 mg/mL the particle size increased from 65 nm up to 150 nm for a FRR of 0.05. The same behavior was found for all other flow rate ratios investigated (Figure 3).

This effect is most likely related to the increasing viscosity of the organic phase, which led to impeded diffusivity of the organic phase into the aqueous phase, and thus to a longer mixing time. Also, it appears that a large number of nuclei are formed and the high concentration of polymer per unit volume promotes particle growth, therefore larger particles were obtained [43]. Using a PLGA concentration of >10 mg/mL resulted in clogging of the mixing channel due to agglomeration. For concentrations less than 1 mg/mL, we were not able to produce monodisperse PLGA NPs; several peaks ranging from 20 nm up to 100 nm were observed in DLS (data not shown). This was supported by a large PDI (>0.7), indicating a polydisperse sample [44]. Furthermore, the presence of very small particles could originate from micelle formation from the stabilizer present, as previously discussed in literature [42].

### Effect of the diameter of the focus channel

**Figure 4:**
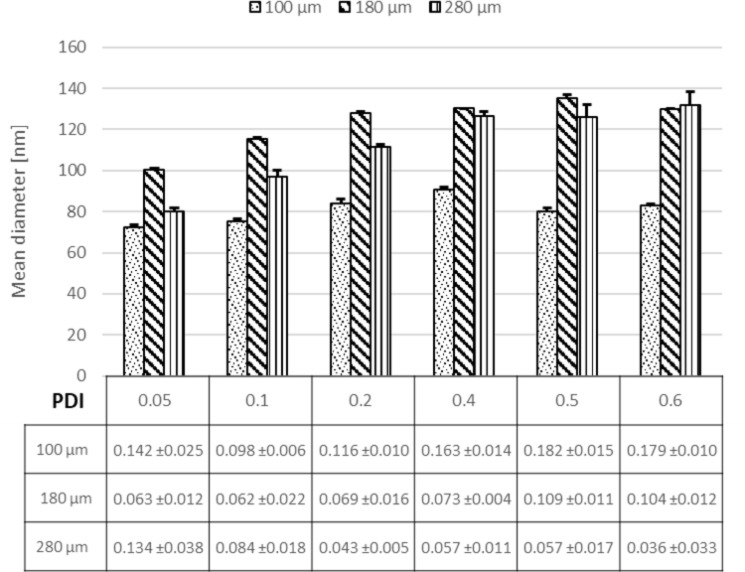
Effect of adjusting the diameter of the focusing channel (100 µm, 180 µm and 280 µm) on NP size and size distribution at different flow rate ratios.

Besides the flow rate, flow rate ratio and polymer concentration, the channel geometry is an important factor impacting on the NP size. This holds true especially for the width of the focus channel in which NP formation takes place. Literature has demonstrated that the key factor to control the NP properties is the mixing time (τ^mix^), which is directly connected to the channel dimensions. The mixing time depends on the geometry as described by [36]:

[2] $$\tau^{\text{mix}}\sim \frac{w_{\text{f}}^{2}}{4D}=\frac{w^{2}}{9D{\left(1+\frac{1}{\text{FRR}}\right)}^{2}}$$

where *D* is the diffusivity of the used solvent, *w*_f_ is the width of the focus channel, *w* is the width of the other channels, and FRR is the flow rate ratio of the organic phase to the aqueous phase. According to this equation, modulating the width of the focus channel *w*_f_ will modulate the flow rate of the organic phase to the aqueous phase. As the focus channels in our microfluidic system can be varied from 20 µm up to 300 µm, we could elucidate the influence of the channel width on the nanoparticle properties. In Figure 4 it can be seen that the NP size was reduced from 120 to 70 nm as a result of modulating the width of the mixing channel from 280 to 100 µm at a 0.05 flow rate ratio due to shortened mixing times. This effect can be clearly seen for the slowest FRR. For larger FRR values, the effect between channel sizes of 180 and 280 µm is no longer evident as the effect of mixing time on particle size has less influence (this behavior is similar to that of a batch reactor [46]). Due to the reduction of the channel diameter, the mixing time was minimized to a FRR of 0.05 (0.27 ms) in contrast to τ^mix^ = 2.19 ms at a FRR of 0.6. Additionally, a focus channel of 50 µm was tested, but this, unfortunately, resulted in a multimodal size distribution of NPs, which is most likely due to the non-stable flow pattern (turbulent flow instead of laminar flow).

### Effect of stabilizer concentration

**Figure 5:**
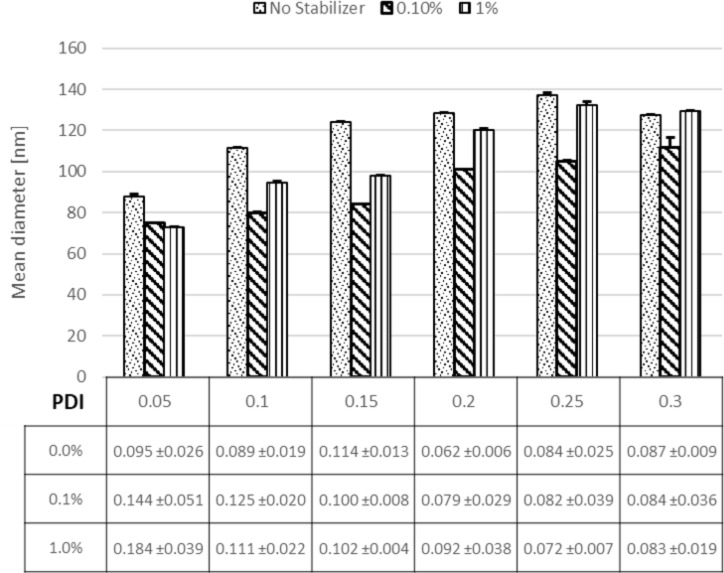
Effect of stabilizer concentration (0.1% and 1% of F68) on NP size and size distribution compared to NPs without stabilizer.

Additionally, to aid the successfully permeation of the NPs through mucus without being trapped, the PLGA NPs can be coated with a muco-inert stabilizer. Using the appropriate stabilizing molecules could reduce the interaction with mucus, and at the same time, foster stability of the colloidal system, thus minimizing NP agglomeration and nucleation growth [40]. Pluronic F68 was proven to be a muco-inert material [11,47] and was therefore chosen as a stabilizer. The addition of Pluronic F68 (1%) resulted in a slight decrease of the NP diameter to ≈70 nm at 0.05 flow rate ratio in comparison to the stabilizer-free (water) NPs with a 90 nm particle diameter (Figure 5).

These observations are consistent with previously presented results with respect to the presence or absence of stabilizers [48]. The slight effect on the size might be due to an increased viscosity of the aqueous phase and thus a prolonged mixing time. Also, the small particle size at low concentrations of Pluronic F68 might be due to the dominating effect of the surface tension over the change in viscosity [49].

The viscosity of low concentrated Pluronic F68 solutions might not be strong enough to play a crucial role in influencing the NP size, although it might aid in stabilizing the surface and to avoid nuclei growth. These results were in accordance with results from the conventional methods indicating a more stable system using stabilizers (data not shown). Overall the size differences are very small and the stability of the colloidal system is improved with higher stabilizer concentrations. Therefore, 1% Pluronic F68 might be the most suitable system.

### Effect of solvent nature and solvent mixture

Drug solubility relies fundamentally on the solvent used. Therefore, assessing the influence of the nature of the solvent on the colloidal properties of our drug carrier would be meaningful. Different solvents were used to elaborate their impact on NP size namely, dimethyl sulfoxide (DMSO), acetonitrile and acetone. We observe no correlation between the viscosity of the used solvent and the final NP size but the Hildebrand solubility parameter (Table 1) correlates with the formation of small NPs [50].

**Table 1:** Properties and miscibility of organic solvents with water according to the Hildebrand solubility parameter.

	*M*_w_ [g/mol]	Density [g/cm^3^]	Molar volume [cm^3^/mol]	Heat of evaporation [J/mol]	Hildebrand solubility parameter [MPa^1/2^]

acetonitrile	41.05	0.786	52.23	33225	24.28
DMSO	78.13	1.1	71.03	52900	26.65
water	18.01	1	18.01	44000	48.03
acetone	58.08	0.784	74.08	31300	19.74

**Figure 6:**
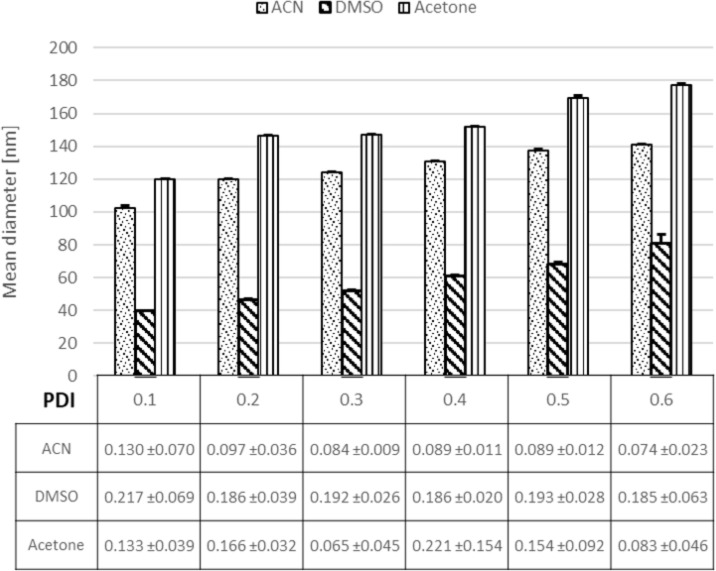
Effect of solvent nature (acetonitrile (ACN), dimethyl sulfoxide (DMSO) and acetone) on NP size and PDI at different flow rate ratios.

When DMSO was used as solvent, the NP size was reduced from 120 to 40 nm at a 0.05 flow rate ratio in comparison to acetone and acetonitrile (ACN), as can be seen in Figure 6.

This data illustrates that the nature of the organic phase plays a decisive role in controlling the diffusion of the organic phase to the aqueous phase, which induces as well a change in the mixing time [50–51].

### Effect of the length of the focus mixing channel

**Figure 7:**
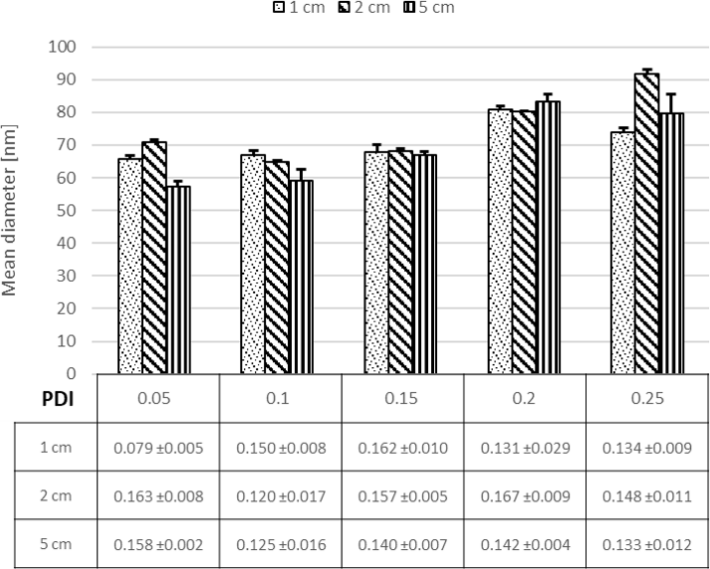
Effect of using different mixing channel lengths (1 cm, 2 cm and 5 cm) on the NP size and size distribution.

Attempts have been made to explain the mechanisms of nanoprecipitation in order to have better control over the kinetics of the colloid formation. To the best of our knowledge, no comprehensive study has presented relevant experimental evidence enabling more insight into the nanoprecipitation mechanism. Our microfluidic system has a unique feature allowing us to select different mixing channel lengths. The impact of the length of the mixing channel was investigated, using 1 cm, 3 cm and 5 cm long mixing channels. Figure 7 shows that the length of the mixing channel has no impact on particle size in our setup, suggesting that possible effects occur at shorter distances after the mixing point.

**Figure 8:**
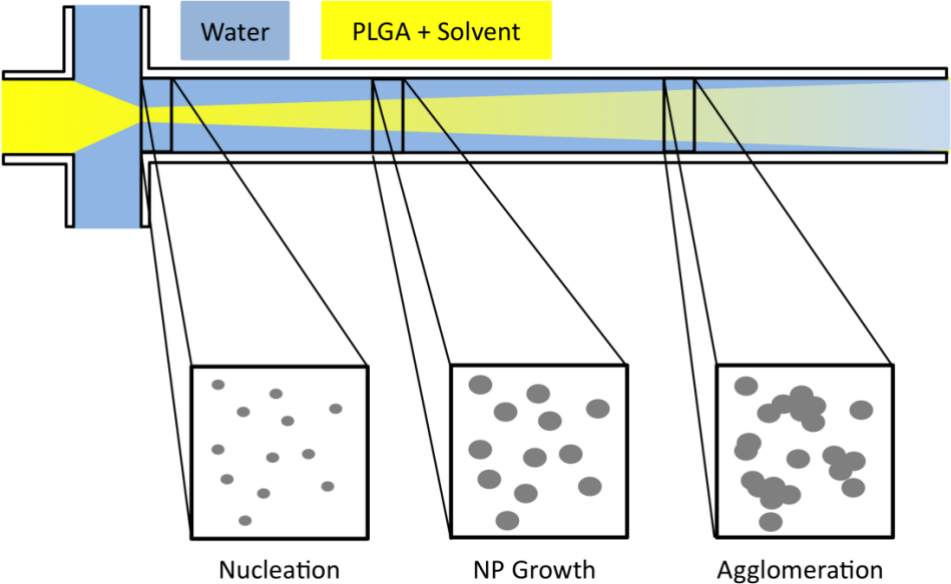
Illustration of nucleation and growth mechanism of nanoprecipitation along the focus mixing channel in a microfluidic system.

It can be seen at the 0.05 flow rate ratio that for all channel lengths the NP diameter was between 50–60 nm. Also for the other flow rate ratios, no influence of the channel length could be observed (Figure 7); only for the highest flow rate ratio, a difference between the different channel lengths could be observed. According to literature, nanoprecipitation is more linked to nucleation and growth, which consists of three stages: nucleation, growth, and aggregation, as illustrated in Figure 8 [52].

Based on the theoretical description of the mechanism, the particle formation is dependent on the time available for growth and agglomeration. Aggregation is assumed to happen after the initial formation and it is assumed to depend on the length of the mixing channel [36]. Therefore, adjusting the length of the mixing channel to only allow for nuclei formation should hinder NP growth, thus ensuring that only the nuclei (small NPs) would be collected.

Overall, the separation of the three stages could be meaningful to understand their respective influence on the colloidal size. We can conclude that on a length scale of >1 cm, the process is already completed.

### Encapsulation of curcumin into PLGA NPs using different techniques

**Figure 9:**
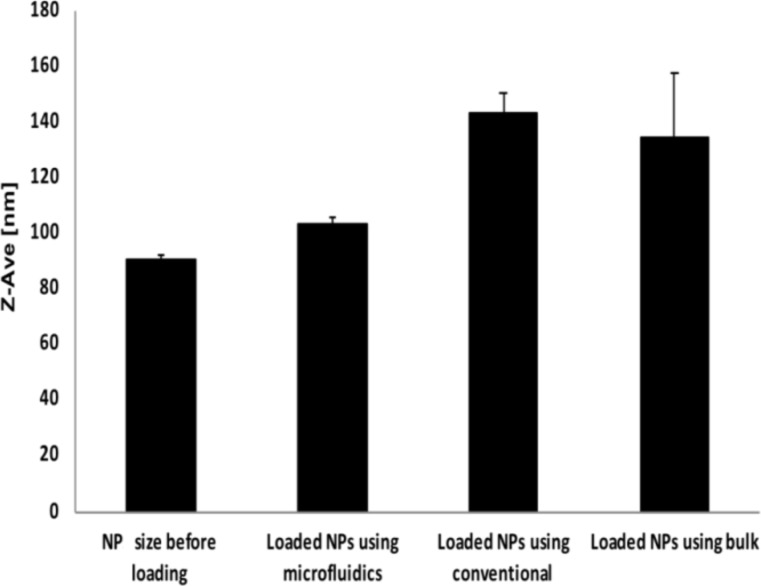
Comparison of the NP size after encapsulation using different approaches, namely the microfluidic system and two benchtop procedures.

Finally, after evaluating the parameters which are relevant to have an influence on the colloidal properties, the incorporation of the drug into the nanocarrier was addressed. The goal was to compare the encapsulation efficiency of curcumin into PLGA NPs while using different approaches (microfluidics, injected by hand (bulk) or using a syringe pump (conventional)). As can be seen from Figure 9, loading curcumin in PLGA NPs using microfluidics resulted in an increase in particle size from 90 nm up to 100 nm.

**Figure 10:**
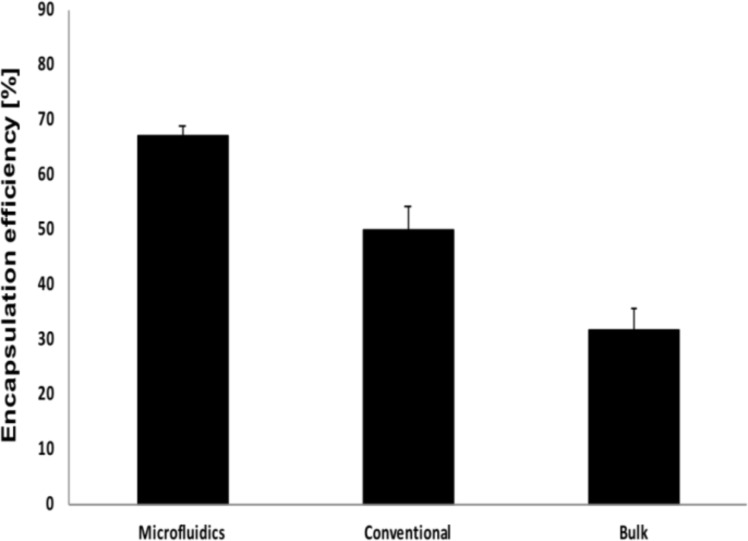
Encapsulation efficiency of curcumin using the different approaches.

In contrast, by loading the PLGA NPs using injection by hand (bulk) or using a syringe pump (conventional), larger sizes were found of up to 145 nm. Thus, microfluidics is an optimal method for producing small, loaded nanoparticles with a good reproducibility and small variability in size. This is most likely due to the ability of microfluidics to mix solutions under laminar flow conditions, ensuring controlled precipitation and short mixing times. Moreover, the microfluidic approach revealed a higher encapsulation of the lipophilic curcumin into PLGA. Using microfluidics, ≈67.15% of the curcumin was encapsulated while the average encapsulation was around ≈50% for the conventional approach (syringe pump) and 30% for hand-injection (bulk) approach (Figure 10), due to the lack of laminar flow conditions and longer mixing time. Higher encapsulation was previously reported for other drugs [36], but for curcumin, no other reports are available to our knowledge.

### Nanoparticle interaction with mucin

**Figure 11:**
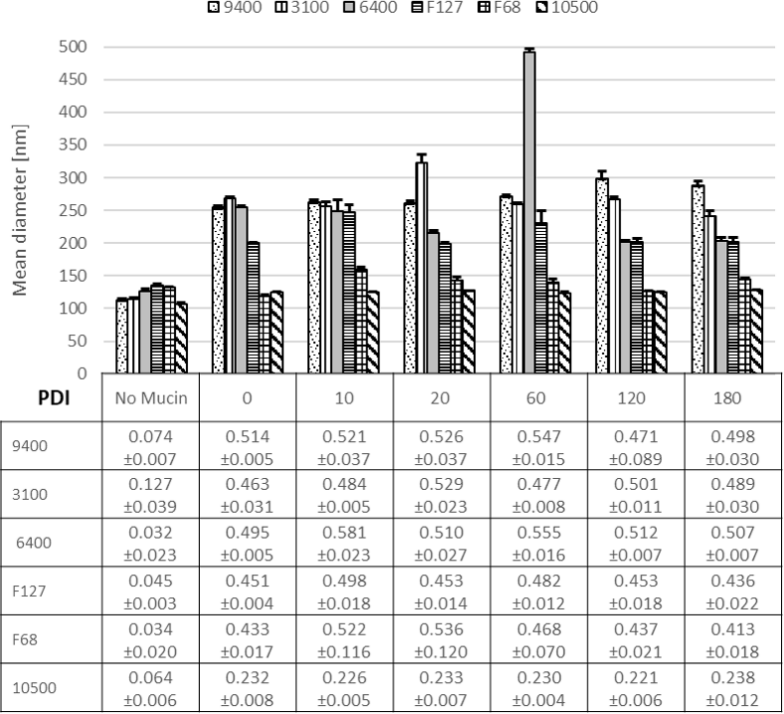
Size and size distribution of nanoparticles stabilized with different types of Pluronic before and after their distribution and interaction with mucin.

The stability of NPs within biological fluids is an essential factor with respect to their potential biological effects [53]. This holds especially true for the interaction of the particles with mucus. To estimate this, a mucin solution was chosen as a simple model for assessing the interaction of different types of surfactant-coated PLGA NPs with mucin [46–47]. The interaction with mucin as a major component for mucus is reflected in size changes and aggregation of the particles. PLGA NPs stabilized with Pluronic F68 or Pluronic 10500 showed no strong interaction with mucin from 0 to 180 min (Figure 11) as no change of size was determined. However, the size distribution for F68-coated particles increased from 0.035 to 0.5. For Pluronic 10500 stabilized particles, the increase was less pronounced, but still observable. In contrast, other types of Pluronic (9400, 3100 and 6400) have directly shown aggregation (from *t*_start_ = 0 min) as obvious from the increase in size (mean diameter up to 400 nm). A considerable shift in PDI from 0.026 to 0.5 was also noticed. The findings could be correlated with the amount of PEG in the polymers, which is more than 50% for Pluronic F68 and Pluronic 10500.

A considerable increase in size and size distribution would hamper the ability of the particles to serve as efficient drug delivery systems. Thus, all stabilizers that do not prohibit agglomeration are not suitable for our application. To investigate if the increase in PDI has a negative influence of the mucus penetration, the diffusion of Pluronic F68 stabilized particles with different sizes was analyzed.

### Permeability of NPs through human pulmonary mucus

The interaction of NPs with mucin is a crude estimate for the interaction with mucus. Besides a specific surface chemistry with a tendency to avoid the interaction with mucins, NPs smaller than the pore size of mucus [54] need to be applied to avoid the size-filtering mechanism [5,10]. A confocal laser scanning microscopy (CLSM)-based set up was used to study the penetration of NPs through pulmonary human mucus [39]. Different sized PLGA NPs (60 nm, 120 nm and 400 nm) were synthesized using microfluidics (fluorescently labelled). NPs in aqueous suspension were added on top of a thin layer of human pulmonary mucus. Then, the penetration kinetics was analyzed by scanning a defined volume at a fixed distance from the objective at different time points.

**Figure 12:**
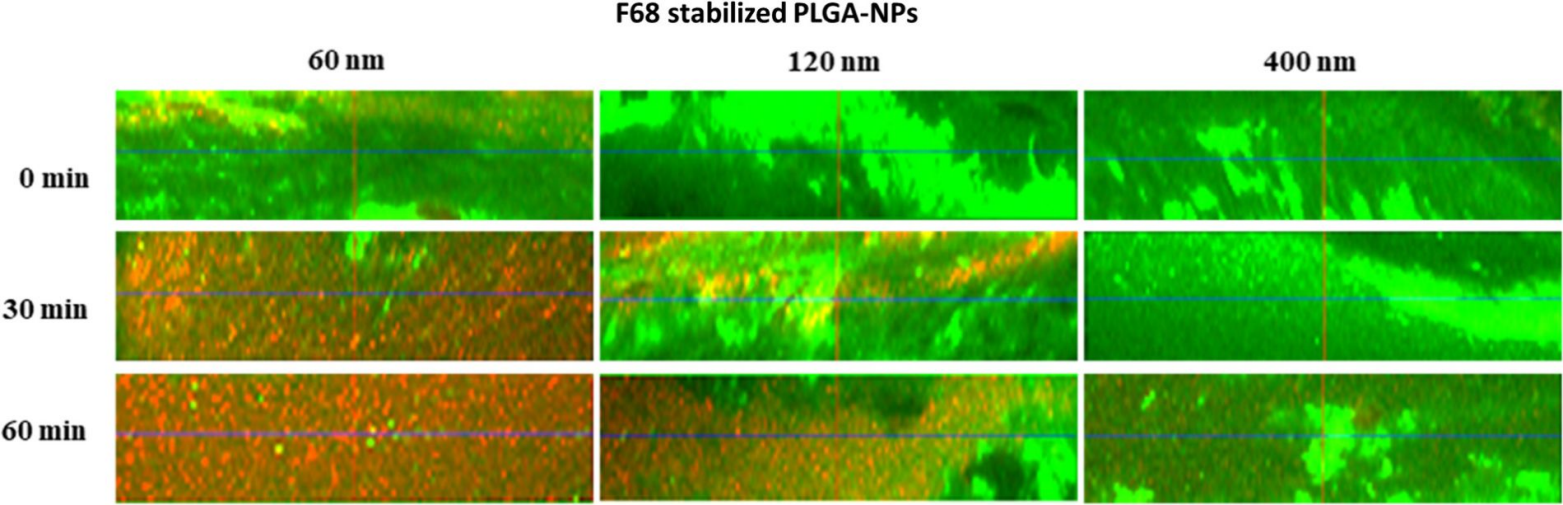
*xz*-micrographs taken in confocal laser scanning microscopy study of the penetration of differently sized F68-stabilized PLGA NPs. Fluorescently labelled (red fluorescence) 60, 120 and 400 nm NPs were imaged along 40 µm of pulmonary human mucus at predetermined time intervals. The mucus was stained with wheat germ agglutinin (green fluorescence).

As illustrated in Figure 12, stabilizer-coated 60 nm PLGA NPs permeated through the human mucus directly when applied.

Penetration was observed up to 1 h after application. In contrast to the 60 nm particles, the 120 nm NPs reached the detection volume only after 30 min, whereas 400 nm particles were hardly observed in the respective volume, indicating no or at least a very slow penetration compared to the two other particle sizes. As all particles were stabilized with Pluronic F68, the stabilizer does not have an influence in this experiment. The only parameter varied was the nanoparticle size, playing a key role for the penetration behavior [10].

## Conclusion

The ability of microfluidics to precisely mix reagents with short mixing times under laminar flow conditions allowed for the generation of monodisperse PLGA NPs of tunable size. Upon varying different factors during the preparation, the results showed that the most dominating influence on the NP size was governed by controlling the mixing time. Furthermore, we could show that the formation of particles is not influenced by a particle growth mechanism due to the diffusion in the mixing channel after a certain channel length (longer mixing times). In addition, the small particles produced in microfluidics were perfectly suited to diffuse through pulmonary mucus as a biological barrier without being immobilized. NPs of approximately 60 nm in diameter have demonstrated improved penetration through pulmonary human mucus in contrast to larger particles of 120 nm and 400 nm diameter. The latter could not reach the observation volume within 1 h, which was attributed to the size-filtering effect. This highly controllable preparation of small particles using microfluidics in combination with a specific muco-inert surface chemistry led to a promising drug delivery system with enhanced mucus penetration. Moreover, a high absolute curcumin encapsulation efficiency of ≈67.15% was obtained using microfluidics. Furthermore, the encapsulation was clearly improved in comparison with conventional bench-top nanoprecipitation methods.

## Materials and methods

### Materials

Porcine mucin, curcumin, rhodamine B (for covalent labelling of PLGA), dichlormethane, dicyclohexylcarbodiimide, 4-(dimethylamino)pyridine and acetonitrile (ACN) were purchased from Sigma-Aldrich (Steinheim, Germany) and poly(lactic-*co*-glycolic acid) (PLGA) (Resomer RG 503 H, 50:50 ratio, average *M*_w_ = 24,000–38,000 Da) was obtained from Evonik Industries (Darmstadt, Germany). Amphiphilic block copolymer Poloxamer (Pluronic F68, F127, 9400, 6200, 3100, 10500 and 6400) was a kind gift from BASF SE (Ludwigshafen, Germany). Pulmonary human mucus was collected by the endotracheal tube method after informed consent from patients (Winterberg Hospital, Saarbrücken, Germany). AlexaFluor-WGA (wheat germ agglutinin) was purchased from Invitrogen (Oregon, USA). All materials employed in the preparation of nanoparticles were of HPLC grade.

### Fluorescence labelling of PLGA

**Scheme 1:**
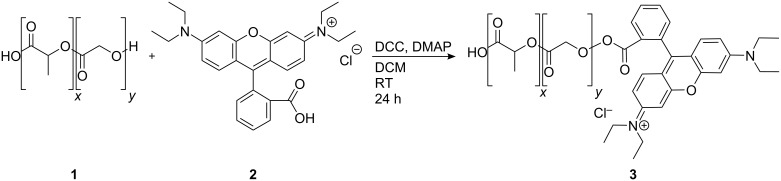
Sketch of the reaction scheme for PLGA labelling with rhodamine B. The carboxy group of the fluorescence dye (**2**) is activated with DCC in water-free DCM and reacts with the terminal alcohol group of PLGA (**1**) under DMAP catalysis to the functionalized polymer (**3**).

In a first step 1.1 equiv of rhodamine B was dissolved in dried dichloromethane (DCM) and activated with 1.5 equiv of dicyclohexylcarbodiimide (DCC, Scheme 1). This solution was stirred at room temperature. Subsequently, a solution with 1 equiv of PLGA and 0.1 equiv of 4-(dimethylamino)pyridine (DMAP), also in dried DCM, were added. The reaction was carried out at room temperature for 24 h. It was quenched by the addition of 1 mL of water. The organic solvent was removed with a rotational evaporator, and the remaining water was discarded. An overview of the concentration of the different components is given in Table 2. The indicated volume of solvents was used for synthesis with 600 mg PLGA and should be adjusted accordingly for other quantities.

**Table 2:** Overview of the concentration of the different components used for labelling.

	*M* [g/mol]	equiv	*n* [µmol]

PLGA RG 502 H	7000–17000	1.0	50
rhodamine B	479.01	1.1	55
DCC	206.33	1.5	75
DMAP	122.17	0.1	5

For the purification step, the polymer was dissolved in 20 mL of acetone and precipitated by adding the same volume of ethanol. The phases were separated by centrifugation at 20,000*g* for 20 min. The colored supernatant was removed and the polymer was obtained as a pink residue. The purification step was repeated for five cycles. After the last centrifugation, the polymer was dried under vacuum.

### Synthesis of PLGA nanoparticles in a microfluidics system

**Figure 13:**
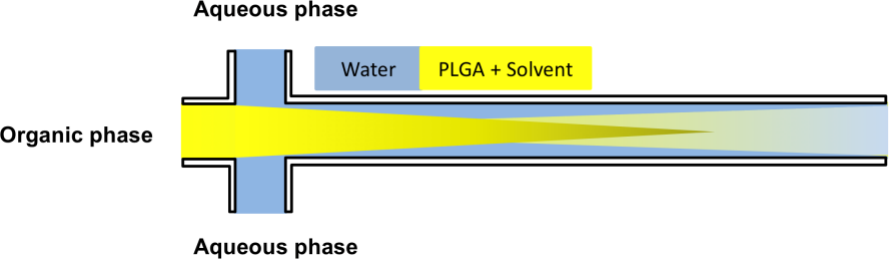
Design and flow pattern of the microfluidic system.

A microfluidic system was assembled using a cross-channel microreactor design, connected via glass capillaries (180 µm ID and 300 OD, Labsmith, Livermore, USA). Monodispersed PLGA NPs coated with Pluronic F68 on the surface were synthesized as illustrated in Figure 13.

In brief, the stock solution of stabilizer containing Pluronic F68 (0.1%) was dissolved in water and injected into the side channels of the microfluidic reactor using a syringe pump (Harvard Apparatus PHD 2000 Syringe, Holliston, USA). In parallel, the organic phase containing 3 mg of PLGA in 1 mL ACN was pumped into the middle channel using another syringe pump (Multi Programmable Syringe Pump, Sarasota, USA). The flow rate ratios (FRR) of the two phases were varied from (0.05 up 1). The two liquids were brought together in the mixing channel and the PLGA started to precipitate and form NPs. The PLGA NP sample was collected from the outlet of the channel. Then the PLGA NP suspension was left overnight under stirring to evaporate the organic solvent. Finally, the PLGA NP suspension was washed twice using centrifugation (30 min at 10,000*g* at 4 °C) and redispersed with Milli-Q water to remove excess stabilizer. The experiments were conducted under the same conditions in triplicate for all formulations.

### Characterization of colloidal PLGA NPs

#### Measurement of size distribution

The colloidal properties of PLGA NPs such as size and size distribution (via the polydispersity index, PDI) were measured utilizing a Zetasizer Nano ZS90 (Malvern Instruments, Malvern, UK) instrument. All measurements were performed after a dilution step to adjust the particle concentration at least in triplicate under the same conditions.

#### In vitro assessment the interaction of NPs with mucin as a simple model

Mucin (1%) was dissolved in water containing 1% NaCl and left overnight under stirring at room temperature to form a kind of artificial mucus (AM). The sample was stored in the refrigerator (4 °C) until use. The AM was incubated with the suspension of PLGA NPs that were stabilized with different types of Pluronic (F68, F127, 9400, 6200, 3100,10500 and 6400) with different block lengths and thus different HLB values in a 1:1 volume ratio at ambient conditions for predefined time intervals. Afterwards, the nano-suspension was centrifuged at 5000*g* for 10 min to separate the NPs from mucin prior to analysis. As a reference, the PLGA NPs before incubation were measured. The properties of the NPs after incubation with AM were measured using DLS to determine whether the NP size increased as a response to strong interactions with AM or if it remain unchanged.

#### The permeability of size-tunable muco-penetrating PLGA NPs through pulmonary human mucus

The permeation of rhodamine B labelled, F68-stabilized PLGA NPs (preparation with 0.1% Pluronic F68) was confirmed by 3D time lapse imaging utilizing confocal laser scanning microscopy (LSM710, Zeiss, Jena, Germany). Each 40 µL of pulmonary human mucus without air bubbles was labelled with 1 µL of AlexaFluor-wheat germ agglutinin. Afterwards, the stained mucus was placed in an imaging chamber made by nail polish on a cover slip resulting in an equally thick mucus layer [55]. At time zero, PLGA NPs were added on top of the mucus and z-stacks within the mucus sample were obtained at constant distance from the bottom of the slide. The permeability of PLGA NPs through mucus was tracked by the change in the fluorescence signal. This approach allowed us to study the size-dependent permeation of PLGA NP through pulmonary human mucus. One day before the experiments, frozen native pulmonary human mucus samples were left to thaw in the refrigerator at 4 °C. Rhodamine B labelled PLGA NPs of 60, 140 and 400 nm diameter were dispersed in Milli-Q water at 0.1% w/v. 5 μL of the nano-suspension was added on top of the mucus. Then, the time-dependent vertical penetration was observed by a 40×/1.1 objective at 37 °C utilizing humidified and temperature-controlled air in an incubation chamber (Zeiss, Jena, Germany) in order to avoid drying. The labelled pulmonary mucus was detected with λ_ex_ = 488 nm at a detection wavelength between 467−554 nm. PLGA NPs were excited at λ_ex_ = 561 nm and the signal was collected between 624–707 nm. The permeability of NPs within mucus was assessed from 0 min up to 1 h after their application. All experiments were made in triplicate.

#### Evaluation of drug encapsulation efficiency using different NP preparation approaches

To evaluate the encapsulation efficiency of curcumin (EE-Cur) into PLGA NPs, the influence of using different techniques was investigated. The EE-Cur after adding the organic phase into the aqueous phase by hand, by using a syringe pump or by using the microfluidic system was compared. In brief, a 9:1 ratio of PLGA to curcumin with a final concentration of 3 mg/mL was dissolved in 1 mL of ACN. Then, the organic phase was precipitated in an aqueous phase containing 0.1% Pluronic F68 as stabilizer. First, to prepare a conventional nanoprecipitation, a plastic syringe was used to inject the organic phase containing (PLGA curcumin) mixture into the aqueous phase by hand (bulk). In parallel to this, the second approach was carried out using a syringe pump (Harvard Apparatus PHD 2000 Syringe, Holliston, USA) with a flow rate setting of 0.1, while in the third approach, an organic phase flow of 20 µL/min and an aqueous phase flow of 200 µL/min were used in a microfluidic setup. The resulting flow rate ratio of 0.1 was used as a standard for NP preparation. All experiments were carried out at least in triplicate.

#### Determination of the encapsulation efficiency of curcumin

For analyzing the EE-Cur, 3 mg of the prepared nanoparticles (PLGA NPs) was dissolved in 2 mL of acetonitrile. 200 µL of each solution was then transferred to a solvent-resistant plate reader plate. The plate was placed in a Tecan plate reader (Tecan, Männedorf, Switzerland) and analyzed using an excitation wavelength of 460 nm and an emission wavelength of 515 nm. A calibration curve for curcumin was prepared using ACN as the solvent. Using the calibration curve, the amount of curcumin inside the sample solution was determined as [Drug encapsulated]. The stock solution for NP preparation contained 0.1 mg of curcumin for each 0.9 mg of PLGA NPs in 10 mL of acetonitrile and was defined as [Drug used]. With this and the analyzed amount of curcumin in the sample solution, the encapsulation efficiency (EE%) can be determined using following formula:

[3] $$\text{EE}\%=\frac{\left[\text{Drug encapsulated}\right]}{\left[\text{Drug used}\right]}*100\%.$$
